# Arsenic Upsets Heartbeat: Possible Early Warning for Cardiovascular Risk

**DOI:** 10.1289/ehp.115-a262a

**Published:** 2007-05

**Authors:** Kris Freeman

Acute arsenic exposure can cause severe heartbeat abnormalities, and chronic exposure has been linked to coronary disease and cancer. Now researchers from Inner Mongolia, China, and the United States have begun to analyze the effects of chronic exposure on the electrical signals that regulate heartbeat **[*EHP* 115:690–694; Mumford et al.]**. They found a correlation between exposure via drinking well water and signal changes associated with arrhythmia and death.

Tens of millions of people worldwide drink groundwater contaminated with naturally occurring arsenic. Through metabolism, the inorganic arsenic found in drinking water is converted to more toxic methylated compounds.

The research team focused on the QTc interval, a specific portion of the cardiac signal that corresponds to the active pumping (systole) phase of the heartbeat. QTc intervals of 0.45 second or longer are associated with cardiac risk.

The team used electrocardiography to measure the QTc interval of 168 men and 145 women from four villages in Ba Men, an area of Inner Mongolia where the drilling of artesian wells in 1980 exposed residents to arsenic. Arsenic exposure was determined through analysis of toenail samples from participants and water samples from their wells. Arsenic exposure was categorized as low (21 μg/L or less), medium (100–300 μg/L), or high (430–609 μg/L).

As exposure to arsenic increased, so did the occurrence of prolonged QTc interval, which was seen in 3.9% of the low exposure group, 11.1% of the medium exposure group, and 20.6% of the high exposure group. Women—who typically have a longer QTc interval than men—were more susceptible to this effect than men. Age, smoking, and pesticide exposure did not affect the association.

The authors suggest that arsenic may affect QTc interval by altering the flow of potassium ions that are involved in cardiac signaling. They write that measurement of QTc interval may be useful in the early detection of cardiovascular risk among individuals exposed to arsenic, as well as in the identification of populations where such risk is high. The team is currently conducting a large follow-up study in the same population. Meanwhile, the Chinese government is helping to install water systems in the Ba Men area that will decrease arsenic exposure.

## Figures and Tables

**Figure:**
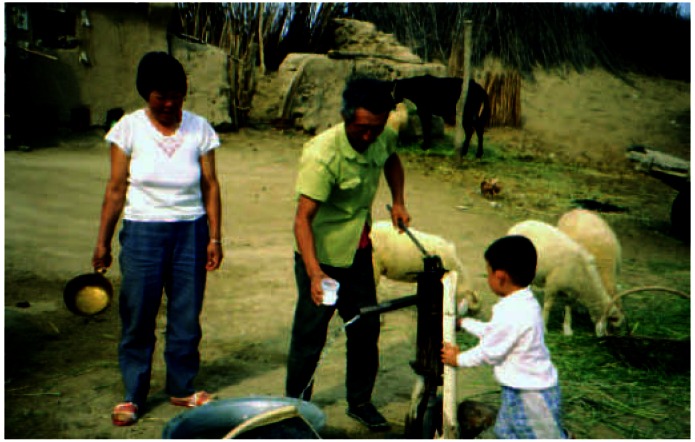
The heart of the exposure Many residents of Ba Men, Inner Mongolia, use artesian well water containing high concentrations of arsenic.

